# Side effects of CT-guided implantation of ^125^I seeds for recurrent malignant tumors of the head and neck assisted by 3D printing non co-planar template

**DOI:** 10.1186/s13014-018-0959-4

**Published:** 2018-02-03

**Authors:** Yuliang Jiang, Zhe Ji, Fuxin Guo, Ran Peng, Haitao Sun, Jinghong Fan, Shuhua Wei, Weiyan Li, Kai Liu, Jinghua Lei, Junjie Wang

**Affiliations:** 1 Department of Radiation Oncology, Peking University Third Hospital, 49 Huayuan North Road, Beijing, 100191 People’s Republic of China; 2 Department of Oncology, Dingzhou City People’s Hospital, Dingzhou, 073000 China; 3 Department of Oncology, Shiyan City People’s Hospital, Shiyan, 442000 China

**Keywords:** 3D printing non co-planar template, ^125^I seed implantation, Side effect, Head and neck carcinoma

## Abstract

**Background:**

For the recurrence of head and neck cancer after operation and radiotherapy, the local control of radioactive seed implantation is good, and it has a certain palliative effect. This study aims to investigate the acute and late side effects of a three-dimentional printing non co-planar template (3D–PNCT) for computed tomography (CT)-guided radioactive ^125^I seed (RIS) implantation in recurrent cancer of the head and neck.

**Methods:**

Between January 2016 and December 2016, forty-two patients with local recurrent malignant tumors of the head and neck received 3D–PNCT-assisted RIS implantation. The prescribed dose was 110–160 Gy. Preoperative planning design, production of individual guide plates, RIS implantation, postoperative dose evaluation, and follow-up were completed for all patients. Side effects in the skin, mucous membranes, blood and spinal cord were evaluated.

**Results:**

All patients underwent surgery successfully. Duration of follow-up was 4–14 (median, of 8.5) months. The activity of a single RIS was 0.34–0.7 (median, 0.6) mCi. The number of RIS was 10–126 (median, 34). The number of implantation needles was 4–31 (median, 11). The mean D2cc (dose to the most exposed 2-cc volume) and D0.1cc (dose to the most exposed 0.1-cc volume) of the skin were 24.9 (7.1–85.5) and 47.5 (9.4–167.2), respectively, whereas those of the spinal cord were 8.4 (4.5–33.3) and 14.2 (13.6–63.0), mucosa were 35.1 (4.2–82.8) and 87.0 (6.6–214.1), parotid glands were 16.2 (12.8–19.7) and 29.8 (26.1–33.4) and those of the trachea were 17.9 (2.5–45.9) and 32.7 (3.9–83.9), respectively. No case had an acute reaction of grade ≥ 3. Three cases had a grade-1 skin reaction. Blood toxicity did not occur, nor spinal-cord injury. Xerostomia was not aggravated than that of before brachytherapy. One case had a grade-3 nerve response.

**Conclusions:**

3D–PNCT-assisted RIS implantation can provide good accuracy for positioning. For local recurrent malignant tumor of head and neck, there were no obvious adverse reactions.

## Background

Implantation of radioactive ^125^I seeds (RIS) is a minimally invasive, safe and effective treatment for malignant tumors. As a radical method for the treatment of early-stage prostate cancer, its long-term efficacy is comparable with that of surgery or external beam radiotherapy (EBRT), but the extent of trauma and number of adverse reactions are much less [1, 2]. RIS implantation also plays an important role in the treatment of tumors of the head, neck, lungs, pancreas, and rectum [3–6]. In terms of recurrent tumors of the head and neck, most patients have already undergone surgery and/or EBRT and chemotherapy for the primary tumor, so treatment is a challenge. For this group of patients, RIS implantation can be good palliative salvage treatment [3, 7, 8].

In recent years, some scholars have taken advantage of computer-aided design and rapid prototyping to a design three-dimensional printing non co-planar template (3D–PNCT) to assist and improve the accuracy of RIS implantation into tumors [9–12]. However, few studies have focused on the side effects of RIS implantation, and there are no reports on the side effects of using a 3D–PNCT. We investigated the side effects of use of a 3D–PNCT combined with computed tomography (CT)-guided RIS implantation for treatment of malignant tumors of the head and neck.

## Methods

### General information

Forty-two patients with recurrent/metastasis of a malignant tumor of the head and neck who received RIS implantation assisted by a 3D–PNCT and CT guidance in our hospital from January 2016 to October 2016 were enrolled. Among them, 26 cases had recurrence of a primary tumor of the head and neck and 16 cases had a metastatic tumor of the neck. Also, 64.3% (27/42) of the patients had undergone head and neck surgery, 85.7% (36/42) of cases had accepted EBRT, and 69% (29/42) of cases had a history of chemotherapy. The Karnovsky Performance Status (KPS) score of all patients was > 70. General information of the patients is shown in Table 1. According to the literature and clinical experiences within our center, 110–160 Gy was the recommended for prescription doses [3, 5, 7, 8, 13–19].

**Table 1:** General characteristics of the 42 patients included in this study

Characteristics	Cases
Sex	
Male	28
Female	14
Age (years)	Median of 61 (29–79)
KPS (points)	Median of 80 (70–90)
Primary disease	
Nasopharyngeal carcinoma	4
Oral cancer	4
Soft-tissue sarcoma of head and neck	3
Hypopharyngeal cancer	2
Oropharyngeal cancer	2
Laryngeal cancer	2
Salivary-gland cancer	2
Thyroid cancer	1
Lymph-node metastasis of unknown primary cancer	1
Esophageal cancer	8
Cervical cancer	3
Lung cancer	3
Breast cancer	1
Colon cancer	1
Staging at first visit	
II	6
III	22
IV	14
Previous surgery	
Once	21
Twice	4
Thrice	1
Four times	1
Previous radiotherapy	
Once	28
Twice	8
Previous cumulative dose (Gy)	
≤ 50	2
50–70	25
70–135	6
Unknown	3
Neoadjuvant/adjuvant chemotherapy	
Yes	29
No	13
Site of implantation	
Head and maxillofacial	16
Lymph node of neck	26

*KPS* Karnofsky performance status

### Preoperative planning design

All patients underwent spiral CT (Brilliance Big Bore; Philips, Amsterdam, the Netherlands) 2 days before surgery. Patients were positioned prone or supine position according to the tumor site. Then, patients were fixed with vacuum pads and marked with a positioning line on the body surface. CT data were transmitted to a Brachytherapy Treatment Planning System (B-TPS). A RIS implantation planning system was designed by the Imaging Center of the Beijing University of Aeronautics and Astronautics (Beijing, China) for preoperative planning. The designed treatment plan on 2D and 3D CT images involved: (i) delineation of the gross tumor volume (GTV) and adjacent organs at risk (OARs); (ii) setting of the prescribed dose and RIS activity; (iii) determination of the needle tract of the implanted RIS (insertion direction, distribution, and depth); (iv) calculation of the RIS number and simulation of the spatial distribution of RIS; (v) calculation of the dose distribution of the target volume and OARs (parotid gland, spinal cord, trachea, mucous membrane, skin, important blood vessels in the neck). We optimized the plan to make the doses of 90% GTV (D90 of GTV) reach the prescribed doses (110–150 Gy) as far as possible while ensuring that the exposure dose to OARs was as low as possible.

### Design and production of individual templates

Depending on B-TPS data, we used 3D images and reverse-engineering software to establish a digital model for the individual template. Coordinates for the spatial points of RIS and needle-tract orientation were incorporated into the model. Then, we used 3D rapid-prototyping equipment with photo-curable resins to print individualized 3D templates. The template contained the superficial anatomic characteristics of the treatment area, positioning markers and simulation of the needle tract.

### Puncture and implantation of ^125^I seeds

**Fig. 1:**
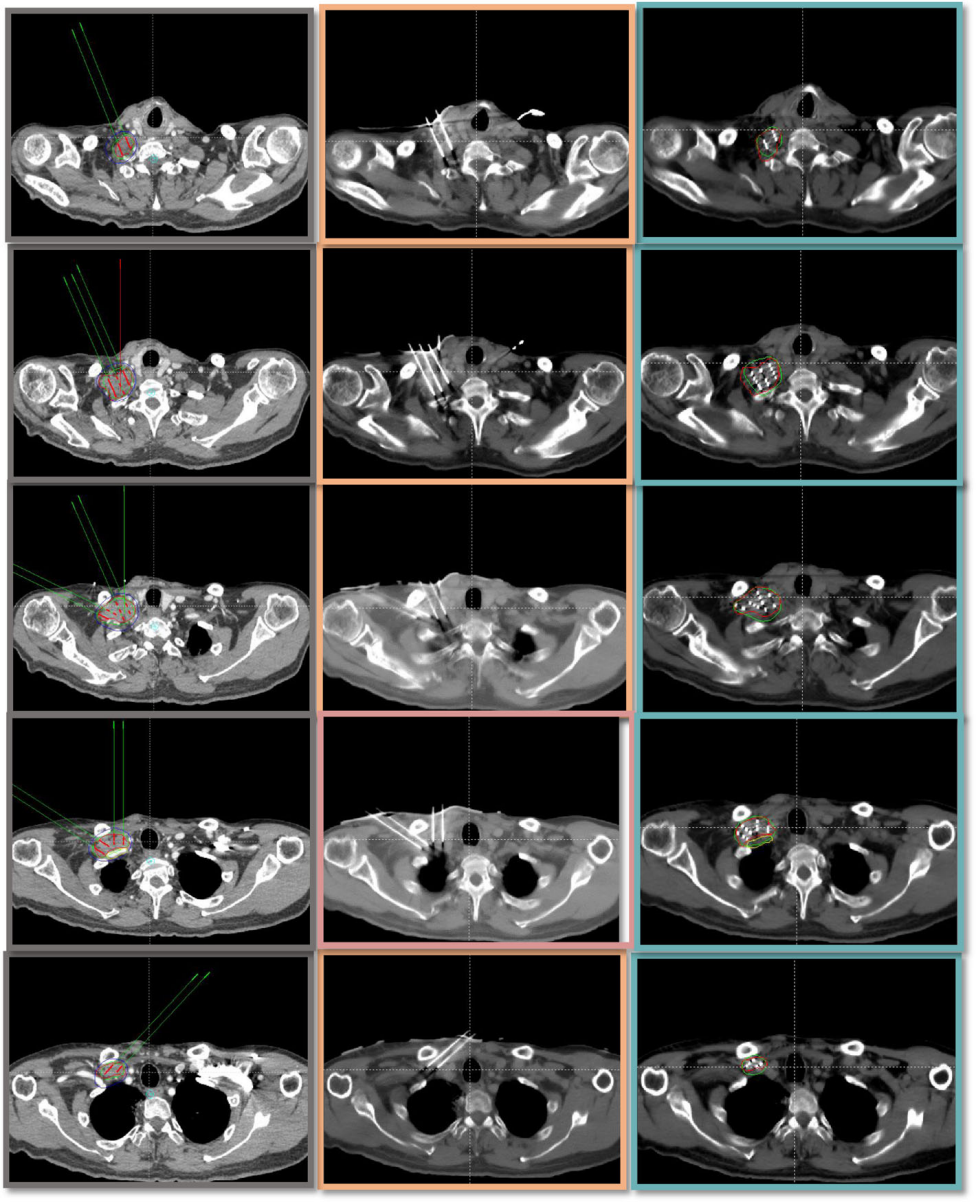
The CT tomographic images of the preimplant and postimplant plan. (Squamous carcinoma of the esophagus yT2N1M1b. Stage IV. Right clavicular lymph node metastasis, EBRT:DT60Gy before). The first column lists all the CT tomographic images of the preimplant plan. The second column shows locations of needles before implantation the RIS. The third column lists the images of the postimplant plan in which includes seeds positions, dosimetric evaluations of GTV and adjacent OARs

Local anesthesia was induced in all patients. The 3D–PNCT was placed on the surface of the treatment area of the patient. The 3D–PNCT was aligned accurately with the outer-contour features, positioning marks, and positioning laser line. Through the guide hole of the 3D–PNCT, we inserted 3 stable needles (Mick Radio Nuclear Instruments, Mount Vernon, NY, USA) percutaneously to pre-planned depths. We carried out CT immediately to validate the locations of stable needles and, if necessary, fine-tuned the intraoperative plan in real time. Finally, depending on preoperative and intraoperative planning, we implanted the RIS in a retrusive manner with a Mick gun (Mick Radio Nuclear Instruments of the USA). Iodine-125 seeds (Tian Jin Sai De Tech Co., Ltd. Atom High Tech Co., Ltd. Chinese Tong Fu Tech Co., Ltd.) with half life of 59.4 days and dose rate constant of 0.965 cGy/(h·U). CT was undertaken immediately after completion of RIS implantation to observe the actual distribution of RIS (Fig. 1).

### Postoperative verification of dosimetry and evaluation of plans

Dosimetric evaluations of GTV and adjacent OARs were based on postoperative CT images.

### Follow-up and research focus

Tumor response was evaluated at 4 weeks and every 2–3 months thereafter. Follow-up involved regular visits and telephone conversations.

The side effects of skin puncture were bleeding, pain, infection, non-union of puncture point, and metastasis due to RIS implantation. The side effects of radiation (which were scored using the toxicity criteria of the Radiation Therapy Oncology Group (RTOG) and the European Organization for Research and Treatment of Cancer (EORTC) were skin injury, mucosal response, spinal-cord injury, peripheral-nerve injury, xerostomia and blood toxicity [20]. Nerve injury was scored by the Common Terminology Criteria for Adverse Events v4.0 [21]. Acute toxicities were evaluated in the first 2 months, and late toxicity were avaluated in 2 months later.

The seed migration, one of specific complications of RIS implantation, was observed during the follow-up period.

### Statistical analyses

With regard to side effects, the chi-squared test was used to analyze significant differences among the grades for the same factor. Related factors were the dose absorbed by the OARs, previous accumulative dose, and preoperative physical condition of the OARs.

## Results

### Treatment and related complications

All forty-two patients completed CT-guided RIS implantation assisted by a 3D–PNCT. The activity of a single RIS was 0.34–0.7 (median, 0.6) mCi. The number of RIS was 10–126 (median, 34). The number of implantation needles was 4–31 (median, 11). All but one episodes of intraoperative pain were tolerable well and disappeared after the surgical procedure. One case had severe intraoperative pain that was barely tolerable, but was relieved postoperatively. Intraoperative bleeding was not obvious. No patients suffered infection at the puncture site after RIS implantation. Skin healing at the puncture site was good except for two cases in which the healing time was delayed for ≈1 week due to exudation. Metastasis due to RIS implantation was not found during follow-up.

### Side reactions of radiation

All the patients were followed up, and the follow-up rate was 100%. The follow-up time was 4 ~ 14 months (median, 8.5 months).

No case had an acute reaction of grade ≥ 3. Three cases had a grade-1 skin reaction: one case had a grade-1 mucosal reaction and two cases a grade-2 reaction. Blood toxicity was not observed.

With regard to late responses, spinal-cord injury did not occur. Xerostomia was not aggravated. One case had a grade-3 nerve response. Preoperatively, this patient had an abnormal sensation with tumor invasion of the brachial plexus and weak lifting force (strength was level 4). Three months after brachytherapy, the tumor had nearly disappeared, but the neurologic symptoms were aggravated, the abnormal sensation was more obvious than before, and the lifting force was weaker (level 3). The nerve reaction worsened from level 2 (preoperative) to 3 (postoperative) (Common Terminology Criteria Adverse Events v4.0). Thus, we speculated that it was more likely to be a radiation-induced nerve injury. However, no evidence could exclude the possibility of tumor invasion because irreversible nerve injury had occurred preoperatively which worsened postoperatively. The severity of the skin reaction was related to the absorbed dose (D0.1cc) (*P* = 0.011). There was little evidence that skin edema and fibrosis were related to the accumulative dose of previous EBRT. One case had nerve injury which might have been related to tumor involvement on nerves. RIS translocation did not occur (Tables 2, 3 and 4).

**Table 2:** Absorbed doses of organs at risks in 3D–PNCT-assisted ^125^I seed implantation salvage treatment for recurrent malignant tumors of the head and neck

	D2cc		D0.1cc	
	Mean (Gy)	Interval (Gy) (standare deviation)	Mean (Gy)	Interval (Gy) (standared deviation)
Skin	24.9	7.1–85.5 (23.1)	47.5	9.4–167.2 (51.2)
Spinal cord	8.4	4.5–33.3 (9.5)	14.2	13.6–63.0 (17.1)
Mucosa^a^	35.1	4.2–82.8 (31.1)	87.0	6.6–214.1 (84.0)
Parotid glands^a^	16.2	12.8–19.7 (49.1)	29.8	26.1–33.4 (51.3)
Trachea^a^	17.9	2.5–45.9 (14.8)	32.7	3.9–83.9 (28.6)

^a^The dose absorbed by this organ was evaluated in some cases. If the tumor margin was < 5 cm and the CT did not include this organ, we did not estimate its dose

**Table 3:** Prevalence of side effects in 3D–PNCT-assisted ^125^I seed implantation salvage treatment for recurrent malignant tumors of the head and neck

	Cases	Percentage
Puncture-related adverse reaction		
Bleeding	0	
Increased pain	1	2.4%
Infection	0	
Skin non-union	0	
Implantation metastasis	0	
Radiation-related adverse reaction		
Early skin reaction		
I	3	7.2%
II	0	
III	0	
IV	0	
Late skin reaction	0	
Early mucosal reaction		
I	1	2.4%
II	2	4.8%
III	0	
IV	0	
Late skin reaction	0	
Blood toxicity	0	
Increased xerostomia	0	
Radiation myelitis	0	
Radiation-based nerve injury	1^a^	2.4%
Movement of radioactive seeds	0	

^a^Tumor invasion of the brachial plexus and weak lifting force (strength level = IV). Three months after surgery, the tumor nearly disappeared, but the neurologic symptoms were aggravated, the abnormal sensation was more obvious than before, and the force was weaker (level III). The nerve reaction worsened from level II (preoperative) to level III (postoperative) (CTC v4.0). Thus, we speculated that it was more likely to be a radiation-based nerve injury

**Table 4:** The chi-squared test of skin and mucosal reactions

	Chi square	*P*
Skin reaction		
D2cc (< 30 vs. ≥30 Gy)	4.1	0.091
D0.1cc (< 60 vs. ≥60 Gy)	10.1	0.011
Previous accumulative dose (< 60 vs. ≥60 Gy)	0.94	0.54
Mucosal reaction		
D2cc (< 30 vs. ≥30 Gy)	0.68	0.41
D0.1cc (< 60 vs. ≥60 Gy)	3.8	0.2
Previous accumulative dose (< 60 vs. ≥60 Gy)	0.94	0.54

D2cc: the dose to 2 cc volume, D0.1cc: the dose to 0.2cc volume

## Discussion

Treatment of recurrent cancer of the head and neck includes reoperation, repeat radiotherapy, and systemic chemotherapy. Because of the previous surgery and/or radiotherapy, the difficulty of reoperation and EBRT increases. In addition, the important anatomic structures of the head and neck, as well as the requirements of functional preservation and cosmetic effect, restrict reoperation and EBRT. However, studies have shown that RIS implantation in the treatment of recurrent cancer of the head and neck has a good palliative effect, good local control, and mild side effects [3, 7, 8].

In recent years, with the development of 3D printing technology, 3D–PNCT-assisted RIS implantation has improved the accuracy of carrying out treatment planning [11, 22–24]. With regard to postoperative verification, the parameters of the conformity index, external index and tumor prescribed doses have coincided closely with that of preoperative planning. Furthermore, application of 3D–PNCT-assisted RIS implantation can be used to predict the doses absorbed by tumors and normal tissues.

Because of the physical characteristics of RIS, the dose absorbed by peripheral tissues declines rapidly, so the side effects are slight. In our previous study on non-template-guided RIS implantation in the treatment of recurrent/metastatic tumors of the head and neck, no side effect was beyond grade 3 [7, 8] and the prevalence of grade-1 and -2 side effects was 21% [8]. With regard to RIS implantation for treatment of metastatic lymph nodes, the prevalence of grade-1 and -1 side effects was 2.8%, and that for grade ≥ 3 was 2.8% [12].

Jinghua et al. [25] undertook surgery with simultaneous RIS implantation. The peripheral matching dose was 60 Gy, patients were not treated with external EBRT, and the prevalence of adverse reactions was 3.6%. Jianguo et al. [26] treated recurrent tumors of the head and neck with RIS implantation, and the prevalence of grade-1 and -2 side reactions (mainly those of skin and mucous membranes) was 24.3%; no case had a side reaction beyond grade-3. Jianguo et al. [27] after adjuvant RIS implantation for patients with oral and maxillofacial tumors with a prescribed dose of 60 Gy, found that 2% of cases had a grade-3 skin reaction and 5.2% of cases had a grade-1 or − 2 skin reaction. Taken together, these reports demonstrated that the side effects of RIS implantation were, in general, low.

In the present study, during follow-up, only one case had a worse nerve reaction that developed from grade 2 preoperatively to grade 3 postoperatively. We speculated that it might have been a treatment-associated side reaction. Reactions in the skin and mucous membrane were all below grade 2, and their prevalence was low (7.2%). Compared with the literature, side effects were improved slightly or to a same extent [3, 7, 8]. According to a preliminary statistical analysis, in three cases with a grade-1 skin reaction, the effect was related to the dose absorbed by the OARs (D0.1cc), whereas the skin reaction was not related significantly to D2cc or the previous accumulative dose of EBRT. The prevalence of side effects was low, besides, there was no significantly difference among the different groups of the correlative factors. In future studies, larger study cohorts will be required.

## Conclusions

Our data show that 3D–PNCT-assisted RIS implantation for the treatment of cancer of the head and neck could guarantee the accuracy of the procedure and provide a reliable absorbed dose of the target volume and OARs. Preliminary results showed no observable side effects. We plan to use larger patient cohorts and clarify its efficacy and safety.

## Abbreviations

^125^I: Iodine-125
3D–PNCT: Three-dimensional printing non-coplanar template
CT: Computer tomography
GTV: the Gross target volume
KPS: Karnofsky performance status
OARs: Organ at risks
RIS: Radioactive ^125^I seed
